# Knowledge, Perceptions, and Practice of Nurses on Surveillance of Adverse Events following Childhood Immunization in Nairobi, Kenya

**DOI:** 10.1155/2016/3745298

**Published:** 2016-12-19

**Authors:** Calistus Wanjala Masika, Harrysone Atieli, Tom Were

**Affiliations:** ^1^Care International, P.O. Box 2360, Kisii, Kenya; ^2^Department of Public Health, School of Public Health and Community Development, Maseno University, Maseno, Kenya; ^3^Department of Medical Laboratory Sciences, School of Public Health and Biomedical Science and Technology, Masinde Muliro University of Science and Technology, P.O. Box 190-50100, Kakamega, Kenya

## Abstract

*Background*. Although vaccines currently approved for routine childhood immunization are safe and effective, frequent adverse events following immunization often cause illnesses and sometimes loss of public trust in immunization programs. Nurses are essential in this surveillance system.* Objective*. To determine nurses' knowledge, perception, and practice towards surveillance of postimmunization adverse events within Nairobi County health centers, Kenya.* Methods*. This is a cross-sectional survey involving nurses (*n* = 274). Data were collected using self-administered questionnaires. Data analysis was performed using SPSS version 20. Differences in proportions of categorical variables were compared between groups using chi-square tests. Binary logistic regression model was used to compute independent predictors of outcome.* Results*. 29.2%, 32.1%, and 45.3% of the respondents had good knowledge, good practices, and good perceptions on AEFI surveillance, respectively. Respondents with diploma or degree nursing training level were 1.8 times and 2.5 times more likely to have good knowledge and good perception in AEFI surveillance, respectively. Nurses with previous AEFI training were 9.7 times and 1.8 times more likely to have good AEFI knowledge and practices, respectively.* Conclusion.* There is a need to train and mentor nurses on AEFI surveillance. Findings of this study will be valuable in informing policy review on childhood immunization programs.

## 1. Introduction

Immunization of infants and young children against serious infectious diseases is the most successful and cost-effective intervention in preventative health care [1, 2]. However, vaccination occasionally leads to undesirable effects including adverse reactions that are referred to as adverse events following immunization (AEFI) [3]. An adverse event following immunization is defined as any untoward medical occurrence which occurs after immunization and which does not necessarily have a causal relationship with the usage of the vaccine [3]. These adverse events are of concern and are believed to be caused by immunization [3, 4]. The commonly encountered adverse events following vaccination include pain at the injection site, swelling, and redness at the site of injection. Others are fever, rash, excessive crying, convulsions, anaphylaxis, encephalitis, drowsiness, or irritability [4]. Although previous studies in China classified occurrence of adverse events into (1) very common (>10%), (2) common (1–10%), (3) uncommon (0.1–1%), (4) rare (0.01–0.1%), and (5) very rare (<0.01%) [5, 6], no studies in Kenya have attempted to identify and classify AEFI occurrence.

The key elements of an effective surveillance system include rapid notification and effective evaluation of the basic information, rapid and effective response, ensuring appropriate outcome of action, and focused responsibility to avoid duplication of efforts [5]. Globally, a range of AEFI surveillance systems have been put in place [7]. For instance, the Global Advisory Committee on Vaccine Safety (GACVS) was established in 1999 to advise WHO on vaccine related safety issues with potential global importance [8]. Many countries have established national monitoring systems to carry out surveillance of adverse events following immunization. 53% of all WHO member countries reported having a national AEFI monitoring system [9]. Previous studies have demonstrated the importance of vaccine safety surveillance [5, 10, 11] though such surveillance and follow-up infrastructure lags behind vaccine development in industrialized countries and is absent in most developing countries including Kenya [1].

In Kenya, passive surveillance of adverse events following immunization is carried out. Although the immunization program in Kenya (KEPI) was started in 1980, there are only three cases of AEFI reported at the national level to date [12]. On the contrary, most countries detect serious AEFI with deaths and hospitalizations [13]. Nurses play a pivotal role in gaining and maintaining public confidence in the safety of vaccines through operational AEFI surveillance [7, 14]. These roles include direct involvement in AEFI detection, investigation, reporting, and management [14]. However, their knowledge, perception, and practices regarding surveillance of adverse events following immunization (AEFI) are understudied [15]. It was unknown whether nurses in Kenya were knowledgeable and trained in AEFI surveillance. Their perception and practices towards AEFI surveillance also largely remained unknown. As such, this study examined the knowledge, perception, and current practices of nurses towards AEFI surveillance.

## 2. Materials and Methods

### 2.1. Study Setting and Participants

This cross-sectional hospital-based study was conducted at health centers in Nairobi County, Kenya, between October 2013 and August 2014. The number of facilities included in this study was distributed proportionally in each of the nine subcounties in Nairobi (Kamukunji, Starehe, Kasarani, Westlands, Dagoretti, Langata, Embakasi, Njiru, and Makadara). There were 50 health centers within the county. Study population comprised staff nurses. Nurses in these health centers routinely administer and monitor vaccines. The vaccines administered are part of the national immunization program comprising BCG, pentavalent, pneumococcal conjugate, polio, and rotavirus vaccines.

### 2.2. Data Collection

Eligible nurses (having worked at least three months at the outpatient and under-five child health departments) from each health center were randomly selected until the required proportionate sample size for that health center was obtained. A total of 274 consenting nurses who met the eligibility criteria were given the questionnaire. Self-administered questionnaire, self-made, was used for collecting data from consenting nurses. This questionnaire included specific questions on nurses' sociodemographic characteristics and their knowledge, perception, and practices towards adverse events following immunization. In order to ensure reliability and reproducibility of the tool, the questionnaire was pretested in one of the health centers within Nairobi County. The health center had characteristics similar to those of other facilities studied.

### 2.3. Statistical Analysis

Data was analyzed using IBM® SPSS version 20.0 (SPSS Inc., USA) software. Results were summarized using frequency tables and pie charts. Knowledge levels were determined using a series of 14 questions on AEFI, its causes, management of AEFI, diagnosis of AEFI, and prevention and reporting of AEFI. The mean (±standard deviation) value was used as the cut-off for defining good (values ≥ mean) and poor (values < mean) knowledge. Perception towards AEFI surveillance was assessed using 7 positive and 7 negative statements on a 5-point Likert scale. The highest possible score was 70 and the lowest possible score was 14. The mean of the cumulative scores was used as the cut-off for good perception (values ≥ mean) and poor perception (values < mean) towards AEFI surveillance. The practice of respondents on AEFI surveillance was assessed using 10 questions. Chi-square test was used to examine differences in proportions between sociodemographic variables and each of the dependent variables (knowledge, perception, and practice). Binary logistic regression tests were used to determine associations between the dependent variables (knowledge, perception, and practice) and independent variables (education, years of experience, AEFI training, and AEFI training modality used).

### 2.4. Ethical Considerations

This study was approved by Kenyatta University Ethics Review Committee (KU/R/COMM/51/204) and was conducted according to Helsinki's declarations. Written informed consent was obtained from all study participants prior to enrolment in the study.

## 3. Results

### 3.1. Baseline Characteristics of the Study Participants

A total of two hundred and seventy-four nurses were recruited into the study. The mean (SD) age of respondents was 41.4 (±9.2) years. Age distribution was as follows: 20–29 years (16.4%), 30–39 years (26.6%), 40–49 years (30.7%), and 50–59 years (26.3%). A majority of the respondents were female (81.0%). Most of the respondents (58.0%) had either diploma or degree level of nursing education as opposed to those with certificate level (42.0%). Respondents had 16.4 (±8.9) mean years of experience as illustrated in Table 1.

### 3.2. Knowledge, Perception, Reporting, and Training in AEFI

Most of the respondents (51.8%) had no prior training in AEFI. Only a few respondents (37.4%) knew the causes of AEFI. Only up to 10.3% of the respondents knew reportable AEFI cases. 25.5% knew that AEFI investigation ought to be commenced within 24 hrs. Less than 40% of the respondents knew how to manage a child with postimmunization anaphylaxis as shown in Table 2.

**Figure 1:**
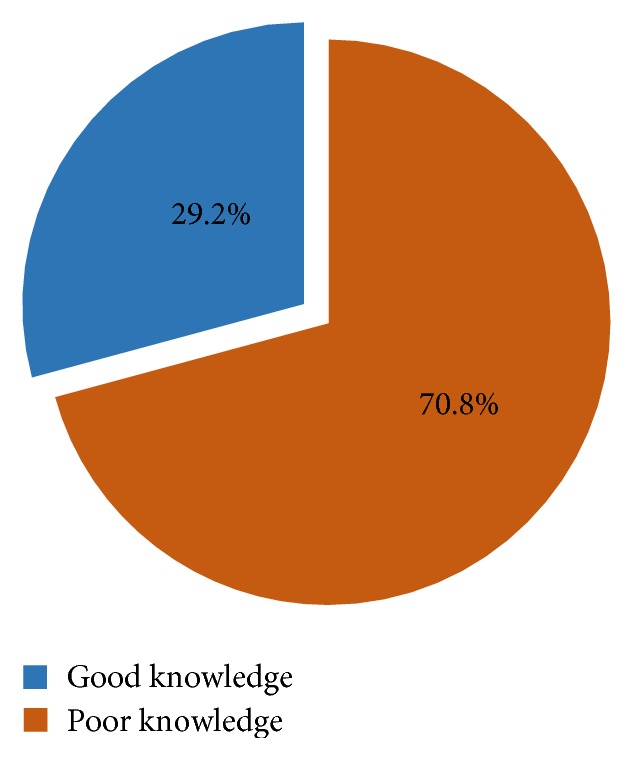
Proportion of nurses with good and poor knowledge on AEFI surveillance.* Good knowledge* refers to the proportion of nurses who have correct responses on surveillance of adverse events following immunization. The opposite is true for poor knowledge.

The overall mean (±standard deviation) knowledge score on causes of AEFI and identification, investigating, managing, and reporting of AEFI was 7.62 (±2.2) out of a maximum of 14. Thus, 194 (70.8%) of the respondents had poor knowledge whereas 80 (29.2%) had good knowledge on AEFI surveillance as shown in Figure 1.

41.9% of the respondents believed reporting an AEFI cannot lead to personal consequences. Less than half (42.3%) of the nurses felt that reporting an AEFI could make them feel guilty about having caused harm and be held responsible for the event. Some respondents (25.2%) felt that the process of reporting an AEFI was long and tedious. However, 77.4% of them acknowledged that nurses play a vital role in diagnosing, reporting, investigating, and managing AEFI. More importantly, 93.8% of the respondents desired to learn more about AEFI surveillance although 9.9% of the respondents were not interested in investigating an AEFI as shown in Table 3.

**Figure 2:**
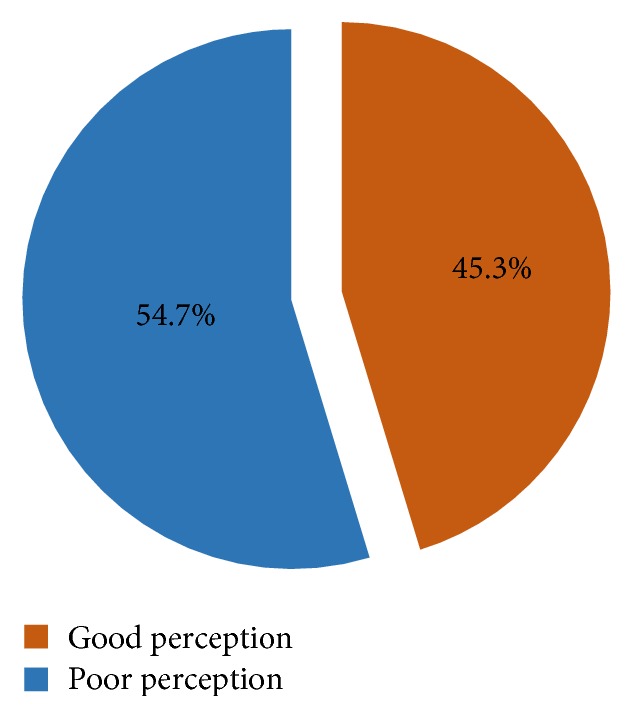
Proportion of respondents with good and poor perception towards AEFI surveillance.* Good perception* refers to the proportion of nurses whose responses on perception questions were deemed supportive to the surveillance of adverse events following immunization. The opposite is true for poor perception.

The mean (±standard deviation) of the cumulative Likert scores on the perception scores for beliefs on detection, reporting, investigating, and managing AEFI was 59.12 (±9.4) out of a maximum of 70. Thus, 124 (45.3%) of the respondents had good perception and 150 (54.7%) of the respondents had poor perception as shown in Figure 2.

Majority of the nurses (76.3%) record vaccine batch numbers and expiry dates during vaccination. Conversely, most nurses (85.8%) did not have an anaphylactic pack with adrenaline in their immunization rooms. Few nurses (32.1%) had ever diagnosed a child with injection site swelling and redness, abscesses, BCG lymphadenitis, convulsion, shock, acute flaccid paralysis, or fever > 40°C. Not many (2.3%) of the nurses had ever participated in AEFI investigation even though 44.5% of them had ever seen an AEFI reporting and investigation form. Surprisingly, only 2.3% of all respondents had ever reported an AEFI as shown in Table 4.

**Figure 3:**
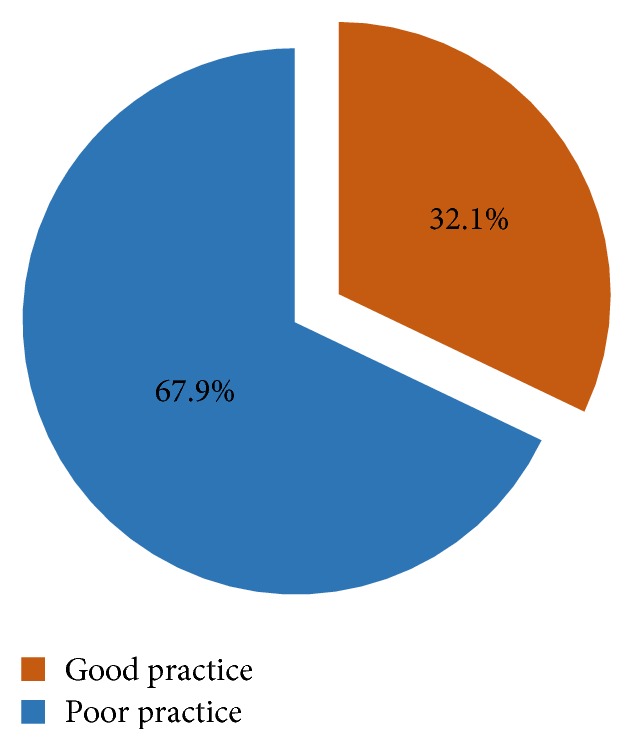
Proportion of respondents with good and poor practice towards AEFI surveillance.* Good practice* refers to the proportion of nurses whose nursing practices advance surveillance of adverse events following immunization. The opposite is true for poor practice.

The mean (±standard deviation) of the cumulative practice scores on practice towards detecting, reporting, investigating, and managing AEFI was 28.45 (±5.7) out of a maximum of 45. Thus, 88 (32.1%) of the respondents had good practice and 186 (67.9%) of the respondents had poor practice towards AEFI surveillance as shown in Figure 3.

### 3.3. Association of Sociodemographic Characteristic and AEFI Knowledge, Practice, and Attitude

Majority of the nurses (77.3%) with previous AEFI training possessed good knowledge in AEFI surveillance (*χ* ^2^: 71.79; *P* < 0.0001). In addition, 56.6% of nurses with diploma or degree nursing education level had good knowledge on AEFI surveillence (*χ* ^2^: 5.23; *P* = 0.022) as shown in Table 5.

Nurses having either diploma or degree nursing training (58.5%) and those with previous AEFI training (61.4%) had good perception towards AEFI surveillance (*χ* ^2^ 13.93, *P* < 0.0001 and *χ* ^2^ 15.82, *P* < 0.0001, resp.) as shown in Table 6.

The practice level towards AEFI surveillance also increased with years of experience since respondents with at least 30 years of experience (75.9%) had good practice (*χ* ^2^ 31.47; *P* < 0.0001). Respondents with previous training in AEFI (65.9%) had good practice compared to those with no previous AEFI training (*χ* ^2^ 5.37; *P* = 0.020) as shown in Table 7.

Additional binary logistic regression analyses revealed that respondents with previous AEFI training were 9.7 times more likely to have good knowledge towards AEFI surveillance [OR: 9.65, 95% CI: 5.55–16.78; *P* < 0.0001]. Similarly, those with diploma or degree level of nursing education were 1.8 times more likely to have good knowledge towards AEFI surveillance [OR: 1.76, 95% CI: 1.08–2.85; *P* = 0.023]. On the other hand, respondents possessing diploma or degree training in nursing were 2.5 times more likely to have good perception towards AEFI surveillance [OR: 2.54, 95% CI: 1.55–4.17; *P* < 0.0001]. Furthermore, respondents aged 30–39 years were 3 times more likely to have good perception towards AEFI surveillance [OR: 3.28, 95% CI: 1.51–7.12; *P* = 0.003]. Respondents with previous AEFI training were 2.7 times more likely to have good perception towards AEFI surveillance [OR: 2.67, 95% CI: 1.64–4.35; *P* < 0.0001]. Nurses practicing in their 30s were 5 times more likely to have good practices towards AEFI surveillance [OR: 5.01, 95% CI: 1.88–13.30; *P* = 0.001]. Those with previous AEFI training were 1.8 times more likely to have good practices in AEFI surveillance [OR: 1.78, 95% CI: 1.09–2.89; *P* = 0.021] as shown in Table 8.

## 4. Discussion

The mean age of nurses in this study is close to that of respondents in a similar study in Nigeria (39.5 years) [16] but much higher than that (33 years) of participants in Zimbabwe [17]. The findings of this study indicating that most of the respondents were female are consistent with findings of similar studies in Zimbabwe and Nigeria [16, 17]. Furthermore, the average years of experience of 16.4 years by respondents in this study are slightly higher than those among respondents in Nigeria (12.2 years) [16]. Respondents in a similar study in Zimbabwe had fewer years of experience (5 years) [17].

The knowledge, perceptions, and practices of nurses towards surveillance of AEFI influence the quality and safety of the vaccination services as well as monitoring and surveillance of AEFI [18]. The overall low knowledge levels on AEFI surveillance recorded by the respondents in this study indicate that nurses in Nairobi County (Kenya) poorly understood AEFI surveillance. The findings of this study are consistent with previous studies reporting low knowledge levels on AEFI surveillance [19]. These findings, however, differ from the higher knowledge levels on AEFI surveillance recorded by nurses in USA [15] and Nigeria [16]. The overall low knowledge on AEFI recorded in this study can be attributed to the consistently low knowledge responses recorded on most of the aspects of AEFI knowledge. Contrastingly, only the AEFI reporting system was recognized by majority of the respondents. This finding is similar to previous studies in the USA showing that most of the nurses knew about the AEFI reporting system [20]. However, other important aspects of AEFI knowledge including immunization error-related reactions that occur during vaccine storage, preparation, handling, and administration were poorly known in spite of their huge contribution to the occurrence of AEFI. This is undesirable since management of AEFI often relies on knowing the cause(s) for appropriate treatment. In Nigeria, majority of the respondents knew various aspects of AEFI [16]. Taken together, these findings on the generally low AEFI surveillance knowledge among nurses working at Nairobi County health centers suggest a need for initial and refresher training on the various aspects of AEFI surveillance.

Association analyses showing that knowledge level of the respondents on AEFI increased with their level of nursing education and previous AEFI training can be explained in part by accrual of knowledge through training exposure. These findings are dissimilar to previous studies in India showing that increasing age and work-related experience determine knowledge levels on drug-associated adverse events [18, 21]. However, these results are similar to previous studies in the United Arab Emirates illustrating that age does not influence knowledge levels on vaccine-induced adverse reactions [21]. Compared to other studies, few nurses in this study had past training in AEFI surveillance [22]. The findings of this study indicating that 48.2% of participants had received AEFI training prior to the study are, however, higher than the 6% recorded in Zimbabwe [17]. This study confirms the need to provide adequate education to nurses, both before and during service. One of the best ways to do this would be to incorporate AEFI surveillance into continuing medical education programs.

The overall proportion of respondents with good perception towards AEFI surveillance in this study constitutes a reasonable fraction of nurses willing to carry out AEFI surveillance. However, more efforts ought to be done to increase the proportion of those with good perception towards AEFI surveillance. The high proportion of respondents ready to learn more about AEFI surveillance as shown in this study will be essential to immunization managers, especially at health center level, to seize this positivity and offer AEFI training opportunities. The findings of this study on nurses' perception towards AEFI surveillance were similar to studies in the United States and Zimbabwe where 18% and 11.5% of the respondents believed that reporting AEFI was not part of their clinical responsibilities, respectively [17, 23]. This scenario emphasizes the need for immunization managers to clearly sensitize nurses on their role in AEFI surveillance. There is a need to reassure nurses that reporting is not meant to be punitive or to apportion blame since half of the respondents in this study believed that reporting AEFI could lead to personal consequences. Although a few nurses cited lack of time as a hindrance to participate in AEFI surveillance, studies in the United States of America indicated higher proportion of nurses citing lack of time [23]. Since having a degree in nursing education increased the likelihood of having good perception towards AEFI surveillance, there is a need to encourage more nurses with certificate nursing education to advance their studies. However, this finding differs from studies in the United Arab Emirates where no difference on perception was observed between nurses with degree and diploma nursing education [21].

Few respondents in this study had ever seen an AEFI reporting and investigation form consistent with findings in a similar study in Zimbabwe [17]. More respondents in a similar study in the United States of America had seen the AEFI reporting and investigation form [15]. This variation could be explained in part by the national sensitization that had occurred in the US a year prior to the study. An AEFI reporting and investigation form is a basic tool essential in carrying out surveillance of AEFI and should always be readily available and accessible to all nurses working in outpatient departments. The finding of fewer nurses in this study who had ever diagnosed a patient with suspected AEFI is consistent with findings in the United States [23]. Postimmunization anaphylactic reactions, though uncommon, are likely to occur during administration of most vaccines. A small proportion of respondents in this study had an anaphylactic pack in their immunization rooms. This finding is comparable to the 33% of the respondents who had pediatric resuscitation equipment in their vaccination rooms in a study in Zimbabwe [17]. This indicates how nurses in this study were unprepared to handle anaphylactic reactions in case they occurred. Even though inaccessibility to AEFI reference guideline materials was cited by majority of respondents in the United States as a hindrance to AEFI reporting [23], only a small proportion of respondents in this study had AEFI guidelines at their workstations. Furthermore, the results of this study are consistent with findings of previous studies indicating low reporting rates among nurses in the United Arab Emirates [21] and the United States [23]. The proportion of the respondents who had ever participated in AEFI investigation was quite low despite the WHO recommendation that health care providers who detect an AEFI ought to report and commence investigations immediately [3]. More than half of the respondents in a similar study in Nigeria had ever reported an AEFI [16]. Compared to the study in the United States, where at least 20% of the respondents had ever reported an AEFI to Vaccine Adverse Event Reporting System (VAERS), most respondents in this study did not know reportable postimmunization adverse events [15]. For instance, only a very small proportion of respondents in this study knew that all injection abscesses, injection site swelling, or redness ought to be reported. On the contrary, AEFI reporting rates among nurses were much higher in studies conducted in Australia [22] and in the United States of America [20]. The findings of this study on recognition of reportable AEFI were similar to those in Australia [22, 23] where there were conflicting views as to which events ought to be reported. There is a need to encourage nurses having many years of experience to mentor nurses with fewer years of experience since good AEFI practice increases with years of experience. The findings of this study indicate that having longer years of experience and previous training in AEFI among respondents is a predictor of good practices in AEFI surveillance. However, in a similar study in Nigeria, there was no statistically significant association between health care worker characteristics and good practices in AEFI surveillance [16].

## 5. Conclusion

Majority of the respondents working at Nairobi County health centers had poor knowledge and poor practice levels on AEFI surveillance. The lowest knowledge levels were in identifying causes of AEFI, how to report an AEFI, and how to investigate and manage postimmunization anaphylaxis. Fear of personal consequences and lack of awareness of nurses' role in reporting an AEFI contributed to poor perception on AEFI surveillance. Most importantly, majority of the respondents were ready to learn more about AEFI surveillance. The practice levels of nurses working at Nairobi County health centers towards AEFI surveillance are poor. Therefore, capacity building of nurse's ability to diagnose, investigate, manage, and report AEFI will go a long way in enhancing AEFI surveillance in Kenya. This can be done through initial and refresher training in AEFI. On-job mentorship on AEFI surveillance can equally be significant.

## Figures and Tables

**Table 1:** Sociodemographic characteristics of study respondents.

Variable, *n* = 274	Frequency (*n*)	Percentage (%)
Age (years)		
Mean (SD)	41.4 (±9.2)	
20–29	45	16.4
30–39	73	26.6
40–49	84	30.7
50–59	72	26.3
Gender		
Male	52	19.0
Female	222	81.0
Level of nursing education		
Certificate	115	42.0
Diploma or degree	159	58.0
Employer		
Nairobi City County	135	49.3
GoK	133	48.5
NGO	6	2.2
Years of experience		
Mean (SD)	16.4 (±8.9)	
0–9	70	25.5
10–19	72	26.3
20–29	103	37.6
30–39	29	10.6
Training in AEFI		
Yes	132	48.2
No	142	51.8

**Table 2:** Knowledge levels of respondents on AEFI surveillance.

Aspects of knowledge on AEFI surveillance, *n* = 274	Freq. (*n*)	%
AEFI as a medical condition is not limited to vaccination only	73	27.8
AEFI can be caused by reconstituted vaccine stored longer than the recommended period; vaccine reaction; inappropriate route or injection technique; vaccines stored beyond expiry date; or contaminated vaccine diluents	102	37.6
Skin at injection site should be stretched during IM injections	99	36.5
Paracetamol and ibuprofen are not used routinely to prevent immunization fever	136	49.6
DHMT is responsible for supervising facilities on AEFI	129	37.8
Adrenaline should not be administered subcutaneously during anaphylaxis	61	22.5
During anaphylaxis, patient's legs are raised above trunk and given oxygen	108	39.4
DPHN receives AEFI reports from facility nurse	163	60.1
AEFI investigation examines operational aspects of the program	99	36.4
Investigation of an AEFI should be commenced within 24 hrs	69	25.5
All injection site abscesses should be reported	28	10.3
Injection site swelling and redness should be reported	22	8.3
Treatment of a coincidental illness falsely attributed as a vaccine reaction should not be delayed until investigations are confirmed	69	25.7
Immunization surveillance aims at early detection and response to AEFI	102	37.2

**Table 3:** Perception of respondents towards AEFI surveillance.

Perceptions on AEFI surveillance	Agree, *n* (%)	Neutral, *n* (%)	Disagree, *n* (%)
Believing that reporting an AEFI cannot lead to personal consequences	115 (41.9)	24 (8.8)	135 (49.3)
Believing that reporting an AEFI, such as injection abscess, will make him/her feel guilty about having caused harm and be responsible for the event	116 (42.3)	32 (11.7)	126 (46.0)
Believing that nurses are reluctant to report an AEFI when they are not confident about the diagnosis	173 (63.1)	40 (14.6)	61 (22.3)
Believing that investigation of AEFI should be done by clinical officers or doctors and not nurses	102 (37.2)	63 (23.0)	109 (39.8)
Believing that poor monitoring of adverse events can cause reduction of immunization coverage	110 (65.1)	24 (9.2)	140 (25.7)
Believing that the process of reporting an AEFI is long and tedious	69 (25.2)	56 (20.4)	149 (54.4)
Believing that reporting and investigating AEFI are none of his/her business	58 (21.2)	11 (4.0)	205 (74.8)
Believing that even if adverse events are reported to DVI/DPHN, no feedback is sent back	32 (11.7)	76 (27.7)	166 (60.6)
Believing that enhancing surveillance of AEFI can help build public trust in immunization program	200 (73.0)	14 (5.1)	60 (21.9)
Believing that nurses play a vital role in diagnosing, reporting, investigating, and managing AEFI	212 (77.4)	0 (0.0)	62 (22.6)
Desiring to learn more about how to diagnose, report, investigate, and manage AEFI	257 (93.8)	0 (0.0)	17 (6.2)
Believing that every nurse working at a health facility should know AEFI	207 (75.5)	6 (2.2)	61 (22.3)
Believing that he/she is always busy and there is no time to report AEFI	136 (49.7)	10 (3.6)	128 (46.7)
Believing that he/she is not interested in investigating or reporting AEFI to DPHN/DVI	27 (9.9)	22 (8.0)	225 (82.1)

Total sample size, *n* = 274. Data are presented as number of subjects and proportions (%). AEFI: adverse events following immunization. DVI: Division of Vaccines and Immunization; DPHN: District Public Health Nurse.

**Table 4:** Practice level of respondents towards AEFI surveillance.

Practice aspects	Yes, *n* (%)	No, *n* (%)
Ruling out contraindications to vaccine(s) in a child prior to administration	230 (83.9)	44 (16.1)
Having an anaphylactic pack with adrenaline in the immunization room	39 (14.2)	224 (85.8)
Informing the caretaker of possible vaccine adverse reactions and how to treat them	155 (56.5)	119 (43.5)
Having ever come across a child with injection site swelling, redness, abscesses, BCG lymphadenitis, convulsion, shock, AFP, or fever > 40°C and diagnosing it as an AEFI	88 (32.1)	186 (67.9)
Reporting detecting an adverse event following immunization	2 (2.3)	86 (97.7)
Participating in AEFI investigation for detected AEFI cases	2 (2.3)	86 (97.7)
Recording vaccine batch number and expiry date during vaccination	209 (76.3)	65 (23.7)
Having ever seen an AEFI reporting and investigation form	122 (44.5)	152 (55.5)
Having AEFI reference guidelines materials at workstation	106 (38.7)	168 (61.3)
Having relevant AEFI specimen transportation containers	69 (25.2)	205 (74.8)

Total sample size, *n* = 274. Data are presented as number of subjects and proportions (%). AEFI: adverse event following immunization; BCG: Bacillus Calmette–Guérin.

**Table 5:** Association between knowledge and respondents' characteristics.

Variable	Good knowledge, *n* (%)	Poor knowledge, *n* (%)	df	*χ* ^2^	*P* value
Age (years)					
20–29	25 (55.6)	20 (44.4)	3	4.86	0.182
30–39	43 (58.9)	30 (41.1)			
40–49	41 (48.8)	43 (51.2)			
≥50	30 (41.7)	42 (58.3)			
Gender					
Female	114 (51.4)	108 (48.6)	1	0.181	0.671
Male	25 (48.1)	27 (51.9)			
Level of nursing education					
Certificate	49 (42.6)	66 (57.4)	1	5.23	**0.022**
Diploma or degree nursing education	90 (56.6)	69 (43.4)			
Years of experience					
0–9	34 (48.6)	36 (51.4)	3	2.17	0.537
10–19	33 (45.8)	39 (54.2)			
20–29	58 (56.3)	45 (43.7)			
≥30	14 (48.3)	15 (51.7)			
AEFI training					
Yes	102 (77.3)	30 (22.7)	1	71.79	**<0.0001**
No	37 (26.1)	105 (73.9)			

Data shown are frequencies (*n*) of subjects and proportions (%). df: degrees of freedom.  *χ* ^2^: Pearson's chi-square. Values in bold are significant *P* values.

**Table 6:** Association between perception of nurses and their characteristics.

Variable	Good perception, *n* (%)	Poor perception, *n* (%)	df	*χ* ^2^	*P* value
Age (years)					
20–29	16 (35.6)	29 (64.4)	3	11.25	**0.010**
30–39	47 (64.4)	26 (35.6)			
40–49	40 (47.6)	44 (52.4)			
≥50	31 (43.1)	41 (56.9)			
Gender					
Female	108 (46.8)	114 (51.4)	1	0.031	0.861
Male	26 (50.0)	26 (50.0)			
Level of nursing education					
Certificate	41 (35.7)	74 (64.3)	1	13.93	**<0.0001**
Diploma or degree nursing education	93 (58.5)	66 (41.5)			
Years of experience					
0–9	27 (38.6)	43 (61.4)	3	6.33	0.097
10–19	36 (50.0)	36 (50.0)			
20–29	52 (50.5)	51 (49.5)			
≥30	19 (65.5)	10 (34.5)			
AEFI training					
Yes	81 (61.4)	51 (38.6)	1	15.82	**<0.0001**
No	53 (37.3)	89 (62.7)			

Data shown are frequencies (*n*) of subjects and proportions (%). df: degrees of freedom.  *χ* ^2^: Pearson's chi-square. Values in bold are significant *P* values.

**Table 7:** Association between practice and respondents' characteristics.

Variable	Good practice, *n* (%)	Poor practice, *n* (%)	df	*χ* ^2^	*P* value
Age (years)					
20–29	23 (51.1)	22 (48.9)	3	5.02	0.170
30–39	43 (58.9)	30 (41.1)			
40–49	57 (67.9)	27 (32.1)			
≥50	38 (52.8)	34 (47.2)			
Gender					
Female	133 (59.9)	89 (40.1)	1	0.64	0.424
Male	28 (53.8)	24 (46.2)			
Level of nursing education					
Certificate	63 (54.8)	52 (45.2)	1	1.29	0.255
Diploma or degree nursing education	98 (61.6)	61 (38.4)			
Years of experience					
0–9	27 (26.6)	43 (61.4)	3	31.47	**<0.0001**
10–19	34 (47.2)	38 (52.8)			
20–29	78 (75.7)	25 (24.3)			
≥30	22 (75.9)	7 (24.1)			
AEFI training					
Yes	87 (65.9)	45 (34.1)	1	5.37	**0.020**
No	74 (52.1)	68 (47.9)			

Data shown are frequencies (*n*) of subjects and proportions (%). df: degrees of freedom. *χ* ^2^: Pearson's chi-square. Values in bold are significant *P* values.

**Table 8:** Logistic regression of knowledge, perception, and practice with sociodemographics.

Characteristic	Wald	OR (95% CI)	*P* value
Good knowledge			
Level of nursing education			
Certificate	Reference		
Diploma or degree nursing education	5.19	1.76 (1.08–2.85)	**0.023**
AEFI training			
No	Reference		
Yes	64.48	9.65 (5.55–16.78)	**<0.0001**
Good perception			
Age			
20–29	Reference		
30–39	8.99	3.28 (1.51–7.12)	**0.003**
40–49	1.72	1.65 (0.78–3.47)	0.189
≥50	0.65	1.37 (0.64–2.96)	0.421
Level of nursing education			
Certificate	Reference		
Diploma or degree nursing education	13.66	2.54 (1.55–4.17)	**<0.0001**
AEFI training			
No	Reference		
Yes	15.51	2.67 (1.64–4.35)	**<0.0001**
Good practice			
Years of experience			
0–9	Reference		
10–19	1.08	1.43 (0.73–2.78)	0.298
20–29	22.72	4.97 (2.57–9.61)	**<0.0001**
≥30	10.43	5.01 (1.88–13.30)	**0.001**
AEFI training			
No	Reference		
Yes	5.33	1.78 (1.09–2.89)	**0.021**

Data shown are odds ratios (OR) of variables with 95% confidence interval (CI). Values in bold are significant *P* values.
